# Profile of Individuals Who Are Metabolically Healthy Obese Using Different Definition Criteria. A Population-Based Analysis in the Spanish Population

**DOI:** 10.1371/journal.pone.0106641

**Published:** 2014-09-08

**Authors:** María Teresa Martínez-Larrad, Arturo Corbatón Anchuelo, Náyade Del Prado, José María Ibarra Rueda, Rafael Gabriel, Manuel Serrano-Ríos

**Affiliations:** 1 Spanish Biomedical Research Centre in Diabetes and Associated Metabolic Disorders (CIBERDEM), Madrid, Spain; 2 Instituto de Investigación Sanitaria del Hospital Clínico San Carlos (IdISSC), Madrid, Spain; 3 Clinical Epidemiology Research Unit, Hospital de La Paz, Madrid, Spain; CAEBi, Spain

## Abstract

**Background:**

Obesity is associated with numerous metabolic complications such as diabetes mellitus type 2, dyslipidemia, hypertension, cardiovascular diseases and several forms of cancer. Our goal was to compare different criteria to define the metabolically healthy obese (MHO) with metabolically unhealthy obese (MUHO) subjects. We applied Wildman (W), Wildman modified (WM) with insulin resistance (IR) with cut-off point ≥3.8 and levels of C- Reactive Protein (CRP) ≥3 mg/l; and Consensus Societies (CS) criteria. In these subjects cardiovascular-risk (CV-risk) was estimated by Framingham score and SCORE for MHO and MUHO.

**Methods:**

A cross-sectional study was conducted in Spanish Caucasian adults. A total of 3,844 subjects completed the study, 45% males, aged 35–74 years. Anthropometric/biochemical variables were measured. Obesity was defined as BMI: ≥30 Kg/m^2^.

**Results:**

The overall prevalence of obesity in our population was 27.5%, (23.7%/males and 30.2%/females). MHO prevalence according to W, WM, and CS definition criteria were: 9.65%, 16.29%, 39.94% respectively in obese participants. MHO has lower waist circumference (WC) measurements than MUHO. The estimated CV-risks by Framingham and SCORE Project charts were lower in MHO than MUHO subjects. WC showed high specificity and sensitivity in detecting high estimated CV risk by Framingham. However, WHR showed high specificity and sensitivity in detecting CV risk according to SCORE Project. MHO subjects as defined by any of the three criteria had higher adiponectin levels after adjustment by sex, age, WC, HOMA IR and Framingham or SCORE risks. This relationship was not found for CRP circulating levels neither leptin levels.

**Conclusions:**

MHO prevalence is highly dependent on the definition criteria used to define those individuals. Results showed that MHO subjects had less WC, and a lower estimated CV-risk than MUHO subjects. Additionally, the high adiponectin circulating levels in MHO may suggest a protective role against developing an unhealthy metabolic state.

## Introduction

Obesity is a major public health problem in recent decades, because it is a key risk factor of type 2 diabetes, cardiovascular disease, dyslipidemia, hypertension, certain cancers [1], [2]. However, a proportion between 20 and 30% of obese individuals may be free of metabolic comorbidities during an unknown variable period of time [3]–[5]. The existence of a metabolically healthy obese (MHO) phenotype was first proposed by Sims in 2001 [3]. Otherwise, there are several prospective studies aimed at investigating MHO subjects are at lower risk of early mortality of any cause, mostly due to cardiovascular disease [6], [7].

Many authors have proposed different diverse definitions of the MHO phenotype according to the presence or absence of specific metabolic abnormalities such as: DM2, dyslipidemia and hypertension in individuals with obesity. Associations and clustering of cardiometabolic risk factors, the clinical phenotype derived from metabolic syndrome (MetS) and the inflammatory biomarkers, have then been widely recently used in categorizing those subjects as metabolically healthy or unhealthy [7], [8].

On the basis of proposed MetS criteria, which have a limited value on the diagnosis of a high cardiovascular risk degree in the clinical setting, diverse authors have suggested in the last decade, the use of the definition of MHO and MUHO phenotypes, as a better clinical cardiovascular risk approach.

This issue is controversial and no clear definition criteria are universally accepted [9].

Therefore the purpose of our work was: Firstly, to compare the different accepted MHO definition criteria (table 1): 1) Wildman (W) [4], 2) Wildman modified (WM) using a cut-off point for HOMA-IR ≥3.8 as described in the Spanish population [10], and levels of C-Reactive Protein (CRP) ≥3 mg/l [11], and 3) MetS in accordance with the Consensus Societies (CS) as reported by Alberti KGMM et al. [12]. Secondly, to describe the estimated CV risk associated with each definition.

**Table 1:** Criteria: Wildman (W), Wildman modified (WM) and Consensus Societies (CS).

WILDMAN	WILDMAN MODIFIED	CONSENSUS SOCIETIES Metabolic syndrome
CARDIOMETABOLIC ABNORMALITIES	CARDIOMETABOLIC ABNORMALITIES	CARDIOMETABOLIC ABNORMALITIES
**1-Elevated blood pressure**: Systolic/diastolic blood pressure ≥130/85 mm Hg or antihypertensive medication use	**1-Elevated blood pressure**: Systolic/diastolic blood pressure ≥130/85 mm Hg or antihypertensive medication use	**1-large waist circumference**:≥94 cm in men and ≥80 cm in women,
**2. Elevated triglyceride level**: Fasting triglyceride level ≥150 mg/dL	**2. Elevated triglyceride level**: Fasting triglyceride level ≥150 mg/dL	**2. Elevated triglyceride level**: Fasting triglyceride level (≥1.7 mmol/l),
**3. Decreased HDL-C level**: HDL-C level <40 mg/dL in men or <50 mg/dl in women or lipid-lowering medication use	**3. Decreased HDL-C level**: HDL-C level <40 mg/dL in men or <50 mg/dl in women or lipid-lowering medication use	**3. Decreased HDL level**: HDL cholesterol level <1.0 mmol/l in men or <1.3 mmol/l in women,
**4. Elevated glucose level**: Fasting glucose level ≥100 mg/dL or antidiabetic medication use	**4. Elevated glucose level**: Fasting glucose level ≥100 mg/dL or antidiabetic medication use	**4. Elevated Glucose level**: Fasting glucose levels ≥5.6 mmol/l or drug treatment
**5. Insulin resistance**: HOMA-IR >5.13 (ie, the 90th percentile)	**5. Insulin resistance**: HOMA-IR ≥3.8 (ie, the 90th percentile)	**5. Elevated blood pressure**: systolic ≥130 mmHg and/or diastolic ≥85 mmHg and/or antihypertensive drug treatment or history of hypertension,
**6. Systemic inflammation**: hsCRP level >0.1 mg/L (ie, the 90th percentile)	**6. Systemic inflammation**: hsCRP level ≥3 mg/L (ie, the 90th percentile)	---------------------
**Criteria for body size phenotypes:**	**Criteria for body size phenotypes:**	**Criteria for body size phenotypes:**
**Normal weight, metabolically healthy**: BMI <25.0 Kg/m^2^ and <2 cardiometabolic abnormalities	**Normal weight, metabolically healthy**: BMI <25.0 Kg/m^2^ and <2 cardiometabolic abnormalities	**Normal weight, metabolically healthy**: BMI <25.0 Kg/m^2^ and <3 cardiometabolic abnormalities
**Normal weight, metabolically abnormal**: BMI <25.0 Kg/m^2^ and ≥2 cardiometabolic abnormalities	**Normal weight, metabolically abnormal**: BMI <25.0 Kg/m^2^ and ≥2 cardiometabolic abnormalities	**Normal weight, metabolically abnormal**: BMI <25.0 Kg/m^2^ and ≥3 cardiometabolic abnormalities
**Overweight, metabolically healthy**: BMI 25.0–29.9 Kg/m^2^ and <2 cardiometabolic abnormalities	**Overweight, metabolically healthy**: BMI 25.0–29.9 Kg/m^2^ and <2 cardiometabolic abnormalities	**Overweight, metabolically healthy**: BMI 25.0–29.9 Kg/m^2^ and <3 cardiometabolic abnormalities
**Overweight, metabolically abnormal**: BMI 25.0–29.9 Kg/m^2^ and ≥2 cardiometabolic abnormalities	**Overweight, metabolically abnormal**: BMI 25.0–29.9 Kg/m^2^ and ≥2 cardiometabolic abnormalities	**Overweight, metabolically abnormal**: BMI 25.0–29.9 Kg/m^2^ and ≥3 cardiometabolic abnormalities
**Obese, metabolically healthy**: BMI ≥30.0 Kg/m^2^ and <2 cardiometabolic abnormalities	**Obese, metabolically healthy**: BMI ≥30.0 Kg/m^2^ and <2 cardiometabolic abnormalities	**Obese, metabolically healthy**: BMI ≥30.0 Kg/m^2^ and <3 cardiometabolic abnormalities
**Obese, metabolically abnormal**: BMI ≥30.0 Kg/m^2^ and ≥2 cardiometabolic abnormalities	**Obese, metabolically abnormal**: BMI ≥30.0 Kg/m^2^ and ≥2 cardiometabolic abnormalities	**Obese, metabolically abnormal**: BMI ≥30.0 Kg/m^2^ and ≥3 cardiometabolic abnormalities

HDL-C: High Density Lipoprotein Cholesterol, HOMA-IR: Homeostasis model assessment of insulin resistance, hsCRP: high sensitivity C Reactive Protein,

BMI: Body Mass Index.

### Design, population

We studied 4,097 subjects from the general Spanish population. Details of recruitment and Study protocols of this population-based survey were previously described [13], [14]. In brief, 5,941 men and non-pregnant women aged 35–74 years, from a targeted population of 496,674 subjects from 21 small and middle-sized towns across Spain were invited to participate. All subjects were sent a personalized letter signed by the principal investigator and the authorities of the Regional Public Health Service, explaining the purpose of the study and requesting volunteering for participation. In case of no response, people were again contacted through telephone up to three times.

Two hundred and fifty-three subjects were excluded as they met one or more of the following exclusion criteria: type 1 diabetes, overt heart or hepatic failure; surgery in the previous year, weight changes >5 Kg within the previous 6 months, and hospitalization by whatever reason at the time of participating in our study.

A total of 3,844 subjects completed the study, 1,754 males and 2,090 females. We used standard procedures adapted from the WHO MONICA protocol [15], approved by our Ethics Committee of Clinic San Carlos Hospital. All participants gave written informed consent. Trained interviewers obtained the following data and implemented a medical questionnaire including: age, sex, parity, menopausal status, family history of diabetes, treatment of diabetes, hypertension, and other relevant chronic diseases.

#### Anthropometric measurements

Included BMI (kg/m^2^) and waist circumference (cm) (WC); the cut-off points previously reported in Spanish population (94.5/89.5 cm for males/females) [16] were considered to define abdominal obesity. Waist measurements were made with a non stretchable fibre measuring tape while study participants were standing erect in a relaxed position with both feet together on a flat surface. WC was measured as the smallest horizontal girth between the costal margins and the iliac crests at minimal respiration. Hip circumference (HC) was measured at the level of the greater femoral trochanters. These measurements were used to compute WC divided by HC [waist-to-hip ratio (WHR)].

The reliability of the anthropometric measurements was established by comparing values obtained by three different interviewers in a sample (n = 3,844) of individuals.

With regards to alcohol intake, subjects were classified in four groups: 1) no alcohol intake (0 g alcohol/day), 2) 1–14.99 g/day, 3) ≥15–29.99 g/day; 4) ≥30 g/day [17], [18]. Smoking habits were recorded as follows: smokers (at least one cigarette per day); non- smokers: never having smoked, and ex-smokers: people who had stopped smoking previous 4 years.

Physical activity was evaluated by asking participants to report their average commitment to various physical activities. We quantified the amount of physical activity by estimating the number of metabolic equivalents (MET) as described (www.cdc.gov). MET estimates were equivalent to the number of hours spent on a particular activity multiplied by a score that was specific for that activity. Subjects were classified in three groups according to their physical activity: low <3 METs; moderate 3.0–6.0 METs; high >6.0 METs.

### Procedures and laboratory studies

After an overnight fasting period, 20 ml of blood were obtained from an antecubital vein without compression. Plasma glucose was determined duplicate by a glucose-oxidase method adapted to an Autoanalyzer (Hitachi 704, Boehringer Mannheim, Germany). Total cholesterol, triglycerides and high-density lipoprotein cholesterol (HDL-C) were determined by enzymatic methods using commercial kits (Boehringer, Mannheim, Germany). Low-density lipoprotein cholesterol (LDL-C) was calculated by the Friedewald formula [19]. A 75-g oral glucose tolerance test (OGTT) was performed and interpreted according to the revised 2003 criteria of the American Diabetes Association [20] Diabetes mellitus was diagnosed when fasting plasma glucose was ≥7.0 mmol/l or 2-h post glucose ≥11.1 mmol/l. Subjects on anti-diabetic medication were also considered to have diabetes. In non-diabetic subjects, fasting plasma glucose of 5.6–6.9 mmol/l was indicative of impaired fasting glucose (IFG) and 2-h glucose of ≥7.8–11.0 mmol/l of impaired glucose tolerance (IGT). Serum insulin concentrations were determined by RIA (Human Insulin Specific RIA kit, Linco Research Inc., St Louis, MO, USA). This assay had a lower detection limit of 2 µU/ml with within and between assay coefficients of variation of <1% and <7.43%, respectively. Cross reactivity with proinsulin was under 0.2%. IR was estimated by homeostasis model assessment of IR (HOMA-IR) using the following formula: fasting insulin (µU/ml) × fasting glucose (mmol/l)/22.5 [21]. In subjects without clinical or biological parameters of IR, the 90^th^ percentile for the HOMA-IR was equal to or greater than 3.8, and this value was considered diagnostic of IR [10].

Leptin and adiponectin serum concentrations were assayed by sensitive/specific RIA as follows: leptin by a highly sensitive RIA (Human Leptin RIA Kit, Linco Research), with a lower detection limit of 0.5 ng/ml to 100 µL, and inter and intra-assays' coefficients of variation were 2%–6% and 3%–7%, respectively. Total adiponectin by a highly specific RIA (Human Adiponectin Specific RIA kit, Linco Research) with a lower detection of 1 ng/ml. Intra and interassay coefficients of variation were 2% and 2.6%, respectively. The cut-off point were for Leptin 9.23 ng/ml (50^th^ percentile) and for adiponectin 9.7 µg/ml (50^th^ percentile).

CRP was measured by using nephelometry high sensitivity C Reactive Protein (hsCRP) as the latest chemistry enhancement for the Image Inmunochemistry System (Beckman). CRPH reagent provides improved low sensitivity to 0.2 mg/l. The intra-assay and inter-assay coefficients of variation for CRP were 3.5% and 3.3% respectively. The cut-off point was CRP ≥3 mg/l [11].

Study subjects were divided into three categories based on BMI: non obese: BMI <25 Kg/m^2^, overweight BMI 25–29.9 Kg/m^2^, and obese: BMI ≥30 Kg/m^2^.

High CV-risk was estimated as ≥20% with the Framingham risk score [22] and ≥5% with the SCORE project for populations at low CV-risk [23].

For the purposes of this study, we used (Table 1) W criteria [4], WM and CS to define MHO [12] as compared to MUHO subjects.

### Statistical analysis

Student t test or ANOVA were used to compare continuous variables expressed as means ± standard deviation (SD). The level of significance was set at 0.05 for all analyses.

Linear regression was used to calculate quantitative variables adjusted for age and sex and their 95% confidence intervals (CI). Otherwise, a logistic regression analysis was performed to evaluate associations of adiponectin, leptin and CRP with MHO. Adjusted Odds Ratios (ORs) and their 95% CI were calculated. The receiver operator characteristic curves (ROC) were conducted to evaluate the performance of the WC, BMI and WHR anthropometric parameters in detecting Framingham risk ≥20% and SCORE risk ≥5% for populations at low cardiovascular disease risk. We used the area under the curve (AUC) with 95% confidence intervals (CI). Associations between different definitions of MHO and high CV-risk (Framingham ≥20% and SCORE ≥5% risk scores) were studied by estimating crude and adjusted ORs using logistic regression models adjusted by sex and age and stratified by BMI categories. Statistical analysis was done using STATA 11 SE.

## Results

The overall prevalence of obesity in our population was 27.5% (n = 1,057) (23.7% in males and 30.2% in females); overweight 45.3% (n = 1,741) (53.1% in males and 38.6% in females) and normal weight 27.2% (n = 1,046) (23.7% in males and 31.2% in females).

The number of obese subjects according to different criteria was as follows: by a) W criteria: 29.11% (n = 1,119), b) WM: 29.37% (n = 1,129), and c) CS criteria: 27.54% (n = 1,059). Among the obese subjects (BMI ≥30 Kg/m^2^), a low number was defined as MHO: a) by W criteria: 9.65% (n = 108); b) by WM: 16.29% (n = 184), and c) by CS criteria: 39.94% (n = 423). The prevalence of MHO was 2.81% by W; 4.78% by WM and 11.02% by CS criteria the whole study population (n = 3,844).

In the total population the prevalence of different categories of glucose status was as follows: a) IFG 16.6% (n = 638), b) IGT 8.3% (n = 319) and c) DM2 7.5% (n = 288).

Tables 2, 3 and 4 include the anthropometric parameters of the whole group of participants, in accordance with respective BMI. Overall, MHO subjects had a significantly lower WC and BMI than MUHO. We also observed significant differences in SBP, DBP, HC between groups. Moreover, there is lower abdominal obesity in MHO than MUHO using any of the three definitions. Only 1.81% of MHO individuals by W criteria showed a normal WC in accordance with cut-off points found for our group in Spanish population (less than 94.5/89.5 cm for males/females) [16], by WM: 2.22% and by CS: 3.03%.

**Table 2:** Basic characteristics and anthropometric parameters in individuals with BMI ≥30 Kg/m^2^ by Wildman criteria, means adjustment by age and sex.

	Metabolically Healthy BMI ≥30 Kg/m^2^	Metabolically unhealthy BMI ≥30 Kg/m^2^	p value
	n = 108	n = 1011	
**Age (Years)** *	52.91 (9.93)	53.59 (9.64)	0.535
**Males (%)**	40.7	38.5	
**Females (%)**	59.3	60.5	
	*-X (95% CI)*	*-X (95% CI)*	
**SBP (mmHg)**	115.45 (112.06–118.84)	137.28 (136.16–138.41)	<0,001
**DBP (mmHg)**	74.18 (72.12–76.24)	85.48 (84.80–86.16)	<0,001
**BMI (kg/m2)**	31.95 (31.50–32.40)	33.46 (33.31–33.61)	<0,001
**WC (cm)**	96.88 (95.41–98.35)	101.17 (100.60–101.74)	<0,001
**WC Males ≥94.5 cm or** **Females ≥89.5 cm (%)**	82.52 (74.81–90.24)	90.44 (88.40–92.48)	0.041
**HC(cm)**	107.08 (105.66–108.51)	109.80 (109.33–110.27)	<0.001
**WHR**	0.91 (0.89–0.92)	0.93 (0.92–0.93)	0.006
	*OR*	*OR*	
**Framinghan** Δ	1	19.88 (6.76–58.48)	<0.001
**SCORE** Δ	1	9.52 (1.84–49.13)	0.007

BMI: Body Mass Index; SBP: Systolic Blood Pressure; DBP: Diastolic Blood Pressure, WC: Waist Circumference; HC: Hip Circumference; WHR: Waist to Hip Ratio;

*Mean ± (SD). -X: Mean, CI: confidence interval.

Δ Logistic regression models independent variables: Framinghan and SCORE risk scores adjustment by age and sex. OR: Odd Ratio.

**Table 3:** Basic characteristics and anthropometric parameters in individuals with BMI ≥30 Kg/m^2^ by Wildman modified criteria, means adjustment by age and sex.

	Metabolically Healthy BMI ≥30 Kg/m^2^	Metabolically unhealthy BMI ≥30 Kg/m^2^	p value
	n = 184	n = 945	
**Age (Years)** *	53.47 (10.04)	53.51 (9.58)	0.965
**Males (%)**	41.22	39.55	
**Females (%)**	58.78	60.45	
	*-X (95% CI)*	*-X (95% CI)*	
**SBP (mmHg)**	121.61 (118.98–124.24)	137.76 (136.59–138.93)	<0,001
**DBP (mmHg)**	78.29 (76.68–79.89)	85.57 (84.86–86.29)	<0,001
**BMI (kg/m2)**	32.14 (31.80–32.48)	33.47 (33.32–33.63)	<0,001
**WC (cm)**	97.39 (96.27–98.50)	101.82 (101.32–102.32)	<0,001
**WC Males ≥94.5 cm or Females ≥89.5 cm (%)**	81.96 (75.95–87.97)	90.72 (88.65–92.79)	0.007
**HC(cm)**	106.87 (105.78–107.96)	109.97 (109.49–110.46)	<0.001
**WHR**	0.91 (0.90–0.92)	0.93 (0.92–0.93)	0.083
	*OR*	*OR*	
**Framinghan** Δ	1	8.54 (4.34–16.79)	<0.001
**SCORE** Δ	1	2.74 (1.05–7.14)	0.039

BMI: Body Mass Index; SBP: Systolic Blood Pressure; DBP: Diastolic Blood Pressure, WC: Waist Circumference; HC: Hip Circumference; WHR: Waist to Hip Ratio;

*Mean ± (SD). -X: Mean, CI: confidence interval.

Δ Logistic regression models independent variables: Framinghan and SCORE risk scores adjustment by age and sex. OR: Odd Ratio.

**Table 4:** Basic characteristics and anthropometric parameters in individuals with BMI ≥30 Kg/m^2^ by Consensus Societies criteria, means adjustment by age and sex.

	Metabolically Healthy BMI ≥30 Kg/m^2^	Metabolically unhealthy BMI ≥30 Kg/m^2^	p value
	n = 423	n = 636	0.086
**Age (Years)** *	52.18 (9.69)	53.82 (9.49)	
**Males (%)**	38.1	38.2	
**Females (%)**	61.9	61.8	
	*-X (95% CI)*	*-X (95% CI)*	
**SBP (mmHg)**	127.67 (126.07–129.27)	138.47 (137.09–139.85)	<0,001
**DBP (mmHg)**	80.99 (80.00–81.97)	86.20 (85.35–87.05)	<0,001
**BMI (kg/m2)**	32.60 (32.40–32.80)	33.59 (33.42–33.76)	<0,001
**WC (cm)**	98.23 (97.58–98.88)	102.31 (101.76–102.87)	<0,001
**WC Males ≥94.5 cm or** **Females ≥89.5 cm (%)**	82.09 (78.56–85.63)	92.32 (90.12–94.53)	<0,001
**HC(cm)**	108.33 (107.68–108.98)	110.26 (109.70–110.83)	<0.001
**WHR**	0.91 (0.90–0.92)	0.93 (0.92–0.94)	0.086
	*OR*	*OR*	
**Framinghan** Δ	1	6.71 (4.29–10.48)	<0.001
**SCORE** Δ	1	2.19 (1.15–4.16)	<0.001

BMI: Body Mass Index; SBP: Systolic Blood Pressure; DBP: Diastolic Blood Pressure, WC: Waist Circumference; HC: Hip Circumference; WHR: Waist to Hip Ratio;

*Mean ± (SD). -X: Mean, CI: confidence interval.

Δ Logistic regression models independent variables: Framinghan and SCORE risk scores adjustment by age and sex. OR: Odd Ratio.

Tables 2, 3 and 4 include a logistic regression model with CV-risk (estimated by Framingham and SCORE risks charts) as the independent variable. Framingham and SCORE risks were associated with increased odds of being MUHO to MHO. ORs tended to be higher using the W definition as compared to the WM and CS definitions.

Tables 5, 6 and 7 include biochemical characteristics of individuals in accordance with their respective BMI adjusted by age and sex. MUHO subjects had different fasting glucose, 2-h post glucose, HDL-C, triglycerides, fasting insulin, HOMA IR and adiponectin levels when compared with MHO subjects. On the other hand, fasting adiponectin levels were significantly higher in MHO than MUH0 subjects. In addition, CRP serum concentrations were lower in MHO vs MUHO, these differences were only statistically different (p<0.05) when WM criteria were used. On the other hand, there is lower abdominal obesity in MHO than MUHO by three criteria.

**Table 5:** Biochemical characteristics and lifestyle in individuals metabolically healthy and unhealthy with BMI ≥30 kg/m^2^ by Wildman criteria, adjustment by age and sex.

	Metabolically Healthy BMI ≥30 Kg/m^2^	Metabolically unhealthy BMI ≥30 Kg/m^2^	p value
	*-X (95% CI)*	*-X (95% CI)*	<0.001
**Fasting glucose (mmol/l)**	4.76 (4.46–5.05)	5.75 (5.64–5.85)	0.001
**Glucose 2 hs (mmol/l)**	5.74 (5.23–6.25)	6.63 (6.45–6.82)	<0.001
**HDL-C (mmol/l)**	1.55 (1.48–1.63)	1.19 (1.17–1.22)	<0.001
**Triglycerides (mmol/l)**	1.00 (0.81–1.19)	1.69 (1.63–1.76)	<0.001
**Fasting insulin (pmol/l)**	68.52 (54.55–82.56)	102.55 (97.82–107.28)	<.001
**HOMA-IR**	2.40 (1.79–3.01)	4.36 (4.15–4.57)	<0.001
**CRP (mg/l)**	2.57 (1.02–4.12)	3.49 (2.99–3.98)	0.269
**Fasting Leptin (ng/ml)**	17.96 (15.85–20.08)	19.55 (18.89–20.20)	0.160
**Fasting Adiponectin (ug/ml)**	12.62 (10.97–14.26)	9.43 (8.67–10.20)	<0.001
**Smoking**			
**Smoker (%)**	23.11 (14.36–31.86)	24.57 (21.60–27.55)	0.756
**Non-smoker (%)**	54.29 (45.38–63.19)	52.92 (49.90–55.94)	0.775
**Former smoker (%)**	22.43 (13.78–31.09)	21.81 (18.97–24.65)	0.893
**Physical Activity**			
**Low (<3 METs) (%)**	33.87 (23.83–43.90)	45.65 (42.17–49.14)	0.029
**Moderate (3.0–6.0 METs) (%)**	51.23 (40.61–61.85)	43.42 (39.95–46.89)	0.170
**High (>6 METs) (%)**	15.05 (7.30–22.80)	10.77 (8.53–13.01)	0.297
**Alcohol intake**			
**0 gr (%)**	48.08 (38.55–57.61)	43.60 (40.42–46.78)	0.381
**0–14.99 gr (%)**	27.89 (18.40–37.39)	21.72 (18.77–24.66)	0.222
**15–29.99 gr (%)**	12.35 (5.31–19.39)	19.03 (16.26–21.79)	0.083
**≥30 gr (%)**	10.96 (4.30–17.63)	15.36 (12.90–17.82)	0.224

BMI: Body Mass Index; -X: Mean, CI: confidence interval HDL-C: High Density Lipoprotein Cholesterol; HOMA-IR: Homeostasis model assessment of insulin resistance; CRP:C-Reactive Protein, METs: Metabolic Equivalents.

**Table 6:** Biochemical characteristics and lifestyle in individuals metabolically healthy and unhealthy with BMI ≥30 kg/m^2^ by Wildman modified criteria, adjustment by age and sex.

	Metabolically Healthy BMI ≥30 Kg/m^2^	Metabolically unhealthy BMI ≥30 Kg/m^2^	p value
	*-X (95% CI)*	*-X (95% CI)*	
**Fasting glucose (mmol/l)**	4.78 (4.56–5.00)	5.80 (5.71–5.91)	<0.001
**Glucose 2 hs (mmol/l)**	6.00 (5.62–6.37)	6.61 (6.42–6.80)	0.003
**HDL-C (mmol/l)**	1.47 (1.42–1.53)	1.18 (1.16–1.22)	<0.001
**Triglycerides (mmol/l)**	1.03 (0.88–1.16)	1.73 (1.66–1.79)	<0.001
**Fasting insulin (pmol/l)**	68.32 (57.81–78.82)	105.61 (100.83–110.38)	<0.001
**HOMA-IR**	2.40 (1.94–2.85)	4.53 (4.31–4.74)	<0.001
**CRP (mg/l)**	1.94 (0.95–2.94)	3.83 (3.30–4.35)	0.001
**Fasting Leptin (ng/ml)**	18.07 (16.64–19.51)	19.60 (18.91–20.29)	0.058
**Fasting Adiponectin (ug/ml)**	11.14 (10.04–12.25)	9.22 (8.47–9.97)	0.040
**Smoking**			
**Smoker (%)**	22.95 (16.26–29.65)	24.15 (21.11–27.20)	0.748
**Non-smoker (%)**	55.78 (48.97–62.60)	55.83 (49.73–55–93)	0.831
**Former smoker (%)**	20.94 (14.49–25.22)	22.28 (19.34–25.22)	0.709
**Physical Activity**			
**Low (<3 METs) (%)**	35.63 (27.91–45.35)	45.73 (42.15–49.32)	0.019
**Moderate (3.0–6.0 METs) (%)**	50.42 (42.33–58.51)	43.51 (39.95–47.08)	0.124
**High (>6 METs) (%)**	14.04 (8.25–19.81)	10.57 (8.2–12.85)	0.273
**Alcohol intake**			
**0 gr (%)**	42.53 (35.37–47.31)	46.67 (43.09–50.24)	0.704
**0–14.99 gr (%)**	27.11 (19.90–34.31)	21.18 (18.18–24.19)	0.136
**15–29.99 gr (%)**	19.78 (13.39–26.18)	18.37 (15.56–21.19)	0.691
**≥30 gr (%)**	4.78 (4.56–5.00)	16.15 (13.58–18.72)	0.032

BMI: Body Mass Index; -X: Mean, CI: confidence interval HDL-C: High Density Lipoprotein Cholesterol; HOMA-IR: Homeostasis model assessment of insulin resistance; CRP:C-Reactive Protein, METs: Metabolic Equivalents.

**Table 7:** Biochemical characteristics and lifestyle in individuals metabolically healthy and unhealthy with BMI ≥30 kg/m^2^ by Consensus Societies criteria, adjustment by age and sex.

	Metabolically Healthy BMI ≥30 Kg/m^2^	Metabolically unhealthy BMI ≥30 Kg/m^2^	p value
	*-X (95% CI)*	*-X (95% CI)*	
**Fasting glucose (mmol/l)**	4.89 (4.77–5.01)	6.00 (5.90–6.11)	<0.001
**Glucose 2 hs (mmol/l)**	5.66 (5.45–5.88)	6.88 (6.67–7.09)	<0.001
**HDL-C (mmol/l)**	1.41 (1.38–1.44)	1.12 (1.09–1.15)	<0.001
**Triglycerides (mmol/l)**	1.11 (1.04–1.19)	1.87 (1.80–1.94)	<0.001
**Fasting insulin (pmol/l)**	79.94 (73.84–86.04)	105.56 (100.32–110.80)	<0.001
**HOMA-IR**	2.89 (2.63–3.16)	4.70 (4.48–4.92)	<0.001
**CRP (mg/l)**	2.87 (2.10–3.63)	3.79 (3.17–4.41)	0.065
**Fasting Leptin (ng/ml)**	18.99 (18.00–19.98)	19.42(18.64–20.19)	0.505
**Fasting Adiponectin (ug/ml)**	10.93 (9.89–11.92)	8.99 (8.03–9.96)	0.011
**Smoking**			
**Smoker (%)**	21.55 (17.59–25.52)	24.77 (21.14–28.40)	0.239
**Non-smoker (%)**	55.22 (51.09–59.34)	52.88 (49.25–56.52)	0.403
**Former smoker (%)**	22.59 (18.60–26.58)	21.33 (17.92–24.74)	0.637
**Physical Activity**			
**Low (<3 METs) (%)**	36.48 (31.81–41.14)	47.32 (41.14–51.50)	<0.001
**Moderate (3.0–6.0 METs) (%)**	48.85 (43.99–53.72)	42.15 (38.02–46.28)	0.038
**High (>6 METs) (%)**	14.69 (11.17–18.22)	10.14 (7.51–12.77)	0.041
**Alcohol intake**			
**0 gr (%)**	43.89 (39.50–48.29)	45.36 (41.50–49.21)	0.621
**0–14.99 gr (%)**	23.74 (19.59–27.90)	20.25 (16.81–23.69)	0.202
**15–29.99 gr (%)**	17.97 (14.24–21.70)	18.46 (15.16–21.75)	0.848
**≥30 gr (%)**	14.10 (10.78–17.41)	15.53 (12.48–18.57)	0.532

BMI: Body Mass Index; -X: Mean, CI: confidence interval HDL-C: High Density Lipoprotein Cholesterol; HOMA-IR: Homeostasis model assessment of insulin resistance; CRP:C-Reactive Protein, METs: Metabolic Equivalents.

Smoking and alcohol intake habits were not significantly different when comparing MHO with MUHO subjects under the three criteria used. Physical activity differed between groups as follows: low grade physical activity (<3 METs) was found for MUHO as compared to MHO subjects no matter which criterion was used. A higher percentage of MHO as compared to MUHO subjects under CS criteria practice moderate (3.0–6.0 METs) and high (>6 METs) physical activity.

**Figure 1:**
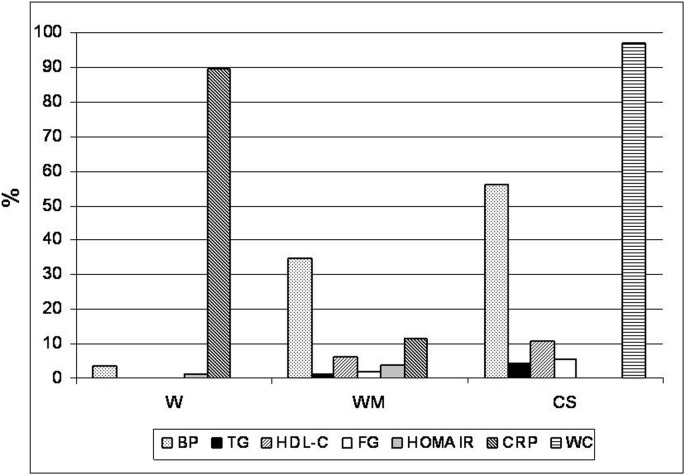
Comorbidities in the MHO subjects by W (Wildman), WM (Wildman modified) and CS (Consensus Societies) criteria according to the data shown in Table 1. BP: Elevated Blood Pressure, TG: Elevated Triglycerides, HDL-C: Decreased HDL-C; FG: Elevated Fasting Glucose, HOMA IR: Elevated Homeostasis Model Assessment Insulin Resistance, CRP: Elevated C Reactive Protein, WC: Large Waist Circumference.

The type and prevalence of comorbidities in the MHO subjects are presented in Figure 1.

Abnormalities included in all three criteria are blood pressure, HDL-C, triglycerides and fasting glucose. Arterial hypertension was the most frequently abnormality found in MHO subjects as defined by CS (56%), followed by WM (35%) criteria.

**Figure 2:**
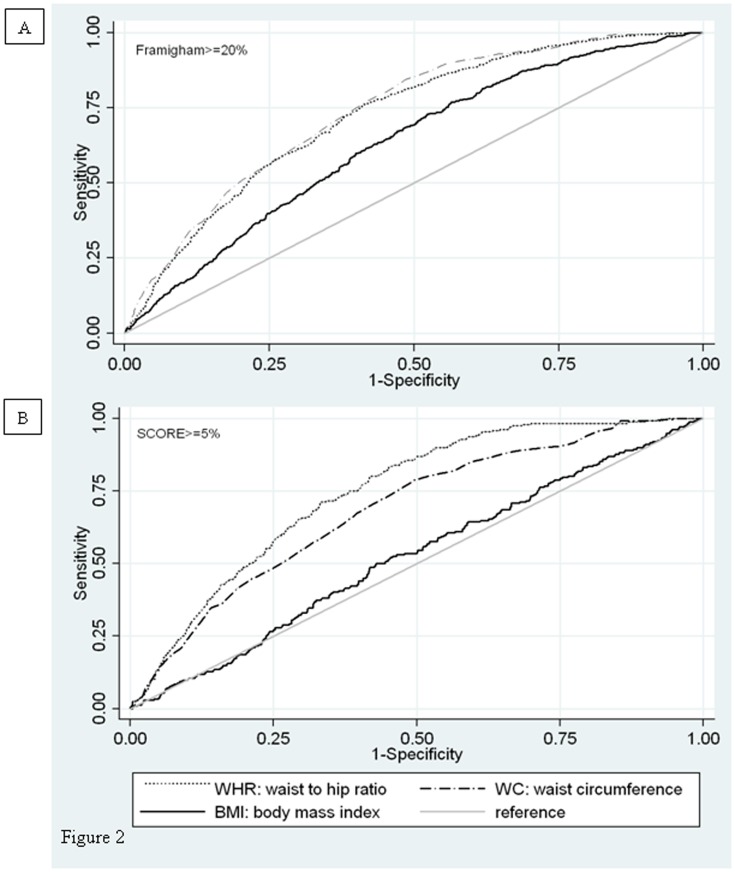
BMI, WC and WHR in detecting Framingham risk score (≥20%) and SCORE Project (≥5%). A.-The AUC was 0.739 (95%CI 0.719–0.759) for WC; 0.724 (95%CI 0.704–0.745) for WHR and 0.631 (95%CI 0.608–0.653) for BMI (p<0.001). B. - The AUC was 0.688 (95%CI 0.655–0.721) for WC; 0.746 (95%CI 0.719–0.773 for WHR, 0.523 (95%CI 0.486–0.561) for BMI (p<0.001).

We obtained ROC curves (Figure 2) for BMI, WC and WHR in detecting Framingham risk score (≥20%) and SCORE Project (≥5%). WC showed high specificity and sensitivity in detecting cardiovascular risk according to the Framingham scale. On the other hand, WHR showed high specificity and sensitivity in detecting high cardiovascular risk according to SCORE as shown in Figure 2. In both cases, all anthropometric measurements correlated with an increased CV risk. Finally, the logistic regression models for W criteria, WM and CS the MHO subjects were associated with elevated levels of adiponectin after adjustment for sex, age, WC, HOMA-IR and CV-risk SCORE project: 1) OR_W(adiponectin)_: 1.04 (95% CI, 1.00–1.07, p = 0.026), 2) OR_VM(adiponectin)_ 1.05 (95% CI, 1.00–1.09, p = 0.015), and 3) OR_CS(adiponectin)_ 1.06 (95% CI 1.00–1.12, p = 0.034).

Adjusted by sex, age, WC, HOMA-IR, and CV Framingham risk score, in all three criteria, MHO subjects were associated with elevated levels of adiponectin: 1) OR_W(adiponectin)_ 1.03 (95% CI, −1.07, p = 0.041), 2) OR_VM(adiponectin)_ 1.04 (95% CI, 1.00–1.08, p = 0.047) and 3) OR_CS(adiponectin)_ 1.08 (95% CI 1.02–1.14, p = 0.002).

Adiponectin is shown as protector of cardiometabolic abnormalities in obese. The risk of developing cardiometabolic abnomalidades is lower in subjects with levels above the median adiponectin. That is the MHO subjects had higher levels than MUHO subjects.

A logistic regression model adjusted by sex, age, WC, HOMA-IR, and CV-risk Framingham risk score or SCORE project for all three definitions criteria used, showed no significant differences in leptin and CRP levels between MHO and MUHO (data not shown).

## Discussion

In the current study on a sample of the Spanish population, the prevalence of MHO according to specific definitions criteria among those with obesity was as follows: W: 9.65%; WM: 16.29%, and CS: 39.94%. Some previously published works reported that among obese subjects, the prevalence of MHO ranged from 3.3% to 43% [4], [7], [24]–[26]. In a cross-sectional analysis carried out by Pajunen P et al. [27], using the CS definition, the MHO individual prevalence was lower (≈13%) than that found in our study (39.94%).

In a 20 year follow up US cohort population, Wildman RP et al. [4] found a 31.7% MHO prevalence using a newly proposed criteria including cut-off point >5.3 (90^th^ percentile). A lower prevalence of about 24% of all obese individuals was found by Hamer M et al. [11] in a representation of the general population study (Health for England and Scottish Health Surveys). In these studies used an adaptation of previous criteria [4], [28].

It is noticeable that among southern European countries very little data on MHO prevalence is available. Most recently in Spain, a well designed epidemiological cross-sectional study including 11,520 individuals by Lopez-Garcia E et al. [5] concluded that MHO subjects represent a 6.5% overall and correspond to 28.9% of obese individuals in the Spanish population aged ≥18 years. Also in this study, 1.8% of subjects MHO showed a normal WC such as it did in our study. Nevertheless a direct comparison between our study and that of Lopez-Garcia et al. is difficult since their population was younger than ours; and the cut-off point they used for HOMA IR and CRP were different. In a population-based prospective follow-up study with 1,051 subjects, by Soriguer F. et al. in Spain [29], the MHO prevalence ranged from 3.0% to 16.9% depending on the set of chosen criteria. In the Cremona Italian prospective study, during a 15 year follow up of 2,011 subjects, Calori G et al. [30] found 11% of MHO individuals among the obese population and 2% within their total study population. The authors used an IR cut-off point for HOMA-IR ≥2.5 as criteria of MHO phenotype, allowing them to compare these results with those from the NHANES III in US [26], where a 6% MHO total prevalence was found. In comparison another Italian population-based study [31] reported a higher (27.5%) MHO prevalence in a cohort of 681 obese individuals living in Rome and surrounding areas. These discrepancies could be related to the anthropometric characteristics of the population, or to regional lifestyle habits which would show a different impact of the typical variety of components (BMI, WC, IR) used in some MHO definitions [6], [32]. It seems, therefore, that the prevalence of the MHO phenotype is highly dependent on the MHO definition and to a certain extent may justify the disparity of results. However, our results show that MUHO individuals tended to have higher WC and HC than MHO counterparts; however, WHR is similar in both phenotypes. This may suggest that MHO and MUHO individuals differ only in terms of the amount and not in the type of adiposity (central vs. peripheral). Therefore, our results may by used to hypothesize that MHO individuals could develop over the years an unhealthy phenotype with further weight gain.

Finally, Van Vliet-Ostaptchouk JV et al. recently compared the defining characteristics of the metabolically healthy obese phenotype across ten population based cohort studies and concluded that there is a “considerable variation in the occurrence of MHO across the different European populations even when unified criteria or definitions were used to classify this phenotype. Further studies are needed to identify the underlying factors for these differences” [33].

### 

#### Lifestyle habits

In our study a lower degree of physical activity was observed in MUHO as compared to MHO individuals depending on the criteria used. However this finding is not consistently reproduced in other published reports [25], [34], [35]. Similarly data on alcohol intake and smoking habits vary widely between different studies [4], [25] including ours.

#### Inflamation

On the other hand, the use of proinflammatory biomarkers as CRP, in the MHO definition criterion, is rarely reported in literature [25], [29]. Marquez-Vidal et al. [36] in a population-based study of 881 obese individuals, found that MHO individuals had lower CRP levels, as did our results in MHO subjects defined by WM criteria.

#### Biomarkers

The potential role of adiponectin must be stressed: as it is one of the major active cytokine molecules produced by white adipose tissue [37] inversely correlated with IR. Otherwise, this cytokine has antiatherogenic and anti-inflammatory properties [38]–[40]. In this context Aguilar-Salinas et al. [24] reported that high adiponectin levels were associated with MHO phenotype. Hence, these Mexican authors proposed the inclusion of adiponectin concentrations in the criteria to define the MHO phenotype. Our own current results support this proposal.

#### Cardiovascular Risk

We found that MUHO individuals defined by three criteria presented higher CV-risk using either the Framingham or SCORE risk than MHO subjects.

Several epidemiological studies has addresed the impact of MHO definition criteria on potential CV-risk [11], [26], with variable results ranging from none to high CV-risk in MHO individuals. On the other hand, Hamer et al. [11] in a large nationally representative sample initially free of CVD, reported that MHO participants did not have increased risk of CVD compared with the metabolically healthy no obese reference group. Our study showed lower CV-risk in MHO than in MUHO subjects. Also, some of the published results in the literature [30], [41] but not all of them [42], [43] are consistent with our finding.

#### Strengths and limitations

A major strength of our study is the high number of carefully phenotyped participants, as well as the availability of new biomarkers such as adiponectin to characterized MHO subjects regardless their definition criteria.

However, there are too some limitations in our work: 1) The cross-sectional design does not allow the establishment of cause-effect relationships. 2) The Framingham risk chart assessment probably overestimates CV- risk in low risk populations such as the Spanish one. Nevertheless, we have tried to attenuate this limitation by using the SCORE project chart, which is widely recommended to estimate CV-risk in low-risk population.

## Conclusions

a) Overall, the prevalence of MHO observed in our population is concordant with some of the previous reported data, in literature. b) MHO and MUHO individuals differ only in terms of amount of adiposity, but not in the type of adiposity (central vs. peripheral). c) Our data show that MHO subjects have a lower estimated CV-risk than MUHO. Likewise, we estimate that amongst the current criteria used to define MHO individuals, WM seems to be the most clinically appropriate one as well as physiopathologically more understandable, since it includes an indirect measure of IR and circulating CRP levels. Insulin resistance linked to obesity is a major risk factor for type 2 diabetes and cardiovascular disease and CRP is an inflammatory marker associated with CV disease in obesity. d) Interestingly enough, we found that high adiponectin circulating levels seem to play a protective role against the risk of developing an unhealthy metabolic state.
